# Deficiency of a brain-specific chemokine-like molecule, SAM3, induces cardinal phenotypes of autism spectrum disorders in mice

**DOI:** 10.1038/s41598-017-16769-5

**Published:** 2017-11-28

**Authors:** Sujin Kim, Boyoung Lee, Jung-Hwa Choi, Jong-Hyun Kim, Cheol-Hee Kim, Hee-Sup Shin

**Affiliations:** 1 Center for Cognition and Sociality, Institute for Basic Science, Yuseong-gu, Daejeon, 34141 Republic of Korea; 2 Basic Science, IBS School, University of Science and Technology, Daejeon, 34113 Republic of Korea; 3 Department of Biology, Chungnam National University, Daejeon, 34134 Republic of Korea; 4 Center for Functional Connectomics, Brain Science Institute, Korea Institute of Science and Technology, Seoul, 02797 Republic of Korea; 5 Laboratory of Cell Death and Human Diseases, Department of Life Sciences, School of Life Sciences, Korea University, Seoul, 02841 Republic of Korea

## Abstract

Chemokines are small secreted signaling proteins produced by a broad range of cells, including immune cells. Several studies have recently suggested potential roles of chemokines and their receptors in the pathophysiology of autism spectrum disorders (ASDs). SAM3 is a novel brain-specific chemokine-like molecule with an unknown physiological function. We explored the relevance of chemokines in the development of ASD in mice, with a focus on SAM3. We generated *Sam3* gene knockout (KO) mice and characterized their behavioral phenotypes, with a focus on those relevant to ASD. *Sam3*-deficient mice displayed all three core phenotypes of ASD: impaired responses to social novelty, defects in social communication, and increased repetitive behavior. In addition, they showed increased anxiety. Interestingly, gender differences were identified for several behaviors: only male *Sam3* KO mice exhibited increased anxiety and increased repetitive behaviors. *Sam3* KO mice did not exhibit changes in other behaviors, including locomotor activities, fear learning and memory, and object recognition memory. These findings indicate that a deficiency of SAM3, a novel brain-specific chemokine-like molecule, may lead to the pathogenesis of ASDs and suggest the possibility that SAM3, a soluble factor, could be a novel therapeutic target for ASD treatment.

## Introduction

Autism spectrum disorders (ASD) are a neurodevelopmental disorder that are mainly characterized by symptoms in social interaction, social communication and repetitive behaviors^1^. In 2013, in the Diagnostic and Statistical Manual of Mental Disorders (DSM)-V, a new guideline for the diagnosis of ASD was defined that integrates previous autistic disorders, such as Asperger’s disorder, childhood disintegrative disorder (CDD), and pervasive developmental disorder not otherwise specified (PDD-NOS) into ASD due to their related signs and symptoms in patients^1^. Moreover, according to the DSM-V, social communication disorder (SCD) was included as a new separate category for patients with disabilities in social communication without the presence of repetitive behaviors^1–3^. Thus, it is critical to identify an appropriate animal model that meets the criteria reflecting the key aspects of human symptoms.

Genetic factors are the most commonly proposed etiology of ASD^4–6^. Despite the numerous studies of gene variations in ASD, no single gene has been identified to confer a risk of autism, which indicates that genetic background may contribute only to the vulnerability of developing brains. Thus, exposing the sensitized brain to environmental insults, such as infection or inflammation during or after pregnancy, may contribute to the development of ASD^7^. Immune dysregulation has recently been reported among children with ASD and their mothers, which suggests potential roles of the immune system in the pathophysiology of the disorder^8^. However, the mechanisms responsible for immune dysfunction and how it is related to ASD development are not understood.

Chemokines, a family of small cytokines, are proteins that regulate the processes of the immune system^9,10^. Several chemokines have recently been implicated in the pathophysiology of ASD and have been suggested as potential biomarkers for ASD^11,12^. In human studies, the plasma levels of macrophage inflammatory protein 1 (MIP-1) alpha, MIP-1 beta and IP-10 have been reported to be highly associated with social behaviors in autistic patients^12,13^. In animal studies, genetic studies using KO mice have also suggested potential roles of chemokines in ASD. For example, mice deficient in CC chemokine receptor 6 (CCR6) displayed higher locomotion, lower anxiety and a reduced preference for social novelty^14^. These studies suggest that chemokines and their receptors may modulate cognition and behaviors related to ASD symptoms.

In this study, our research group first isolated novel functional genes in zebrafish using insertional mutagenesis. Eight members of the zebrafish chemokine-like gene family were identified, which we named the Samdori (Sam) family. We determined that their sequences are highly conserved with respect to the 5 sequences referred to as the FAM19A (TAFA) family sequences in the mouse and human, which have unknown functions. Importantly, these genes are exclusively expressed in the central nervous system with distinct expression patterns in the mouse^15^. Among these genes in the mouse, one gene, Sam3, is predominantly expressed in specific brain regions, including the hippocampus, the medial habenular nucleus, the posterior part of the thalamus, and the pars tuberalis^16^. *Sam3* is distantly related to MIP-1 alpha in sequence, a key inflammatory molecule considered an ASD risk factor^15,16^. Furthermore, a genome-wide analysis of the copy number variation (CNV) identified a *SAM3* gene deletion in an autism patient^17,18^, which raised the possibility that a lack of *SAM3* may play a role in ASD development. To validate the human deletion study and elucidate the role of SAM3 in ASD pathophysiology, we generated *Sam3* KO mice using the transcription activator-like effector nucleases (TALEN) method. To determine whether SAM3 is involved in ASDs, we performed several behavior assays related to the symptoms of ASDs. *Sam3* KO mice exhibited all three core symptoms that constitute the criteria for ASD diagnosis, which suggests that SAM3 plays a preventive role in ASD development.

## Results

### Generation of Sam3 knockout (KO) mice

**Figure 1:**
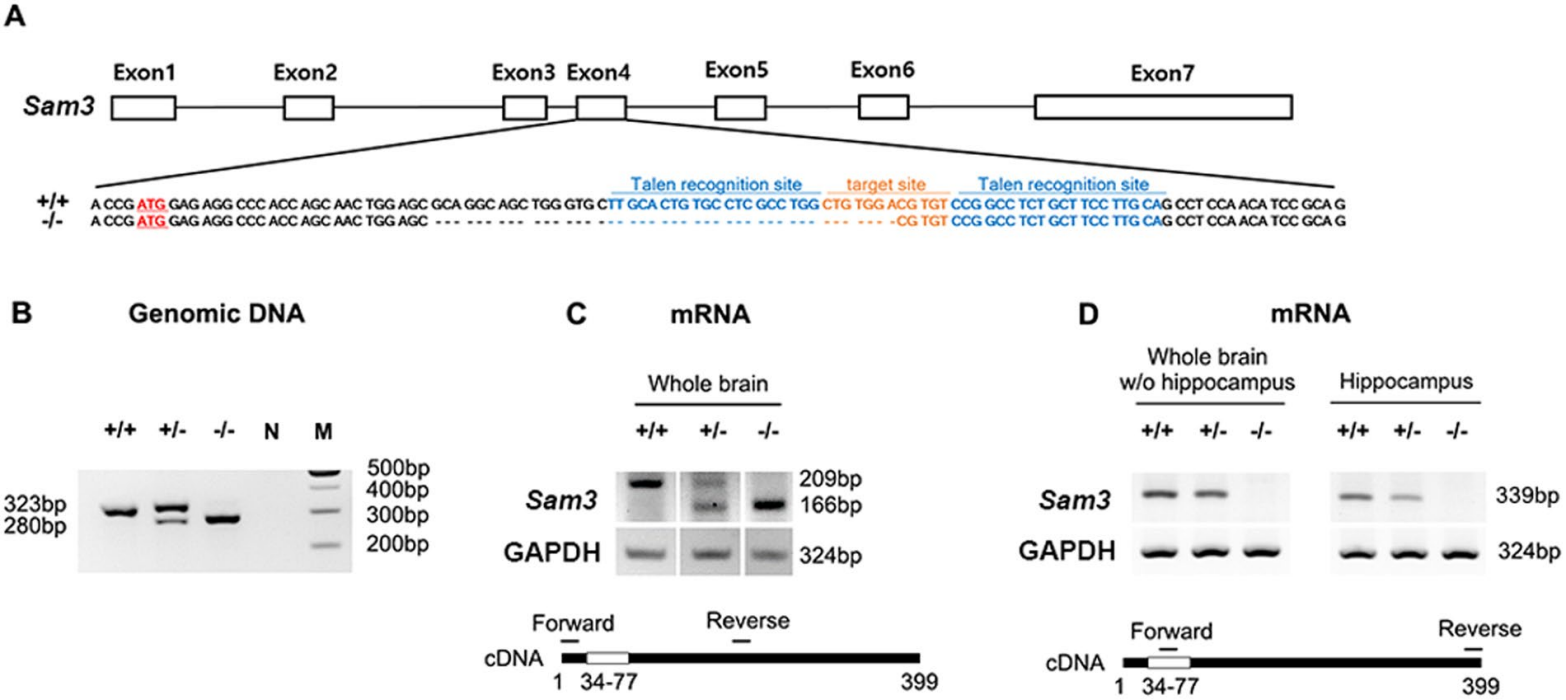
Generation of *Sam3* gene KO mice by TALENs. (**A**) Schematic representation of the genomic structure of the mouse *Sam3* gene. The blue character sequences indicate the positions recognized by the TALEN repeat domains. The targeting sequences are highlighted in orange. The start codon is highlighted in red. “−” denotes deleted nucleotides. (**B**) Gel electrophoresis of the PCR products of SAM3 genomic DNA. (**C**,**D**) RT-PCR analysis of SAM3 mRNA expression from the brains of wild-type mice and heterozygous and homozygous mutants using two different pairs of primers.

A TALEN-mediated KO for the SAM3 gene was produced by targeting exon 4, which contains the initiation codon ATG. A 43-bp deletion from this genomic region of SAM3 was confirmed by sequencing, which resulted in a frameshift mutation with a premature stop codon (Fig. 1A). We confirmed the deletion in genomic DNA via PCR using the primer pair as described in the Materials and Methods: wild-type (323 bp), heterozygous (323 bp, 280 bp) and homozygous KO mice (280 bp) (Fig. 1B). The mRNA expression was subsequently assessed via RT-PCR of whole brain lysates obtained from the mice. Two primer pairs were used for RT-PCR as described in the Materials and Methods. One pair was designed to assess the truncated mRNA, which amplified 209 bp for the wild-type and 166 bp for the mutant. The heterozygous mutant showed both bands (Fig. 1C). The other pairs were designed to confirm the sequence deletion in exon 4; thus, the forward primer was located in the deletion site. No amplified mRNA was detected in both the whole brain lysates without the hippocampus and the hippocampus only lysates of the *Sam3* KO mice (*Sam3* ^−/−^), thus confirming the genomic deletion in exon 4 (Fig. 1D). Furthermore, reduced amplification of the transcripts was identified in the heterozygous *Sam3* ^+/−^ mice (Fig. 1D). We also validated the genomic deletion in female mouse brain (Supplementary Fig. S2). These results confirmed the 43-bp deletion of *Sam3* in both genomic DNA and mRNA. In addition, we tested compensatory effects of other Sam family in Sam3 KO mice. To test that, we performed a quantitative RT-PCR (qRT-PCR). The results showed that there are no compensatory effects of other Sam family in Sam3 KO mice at the transcript level (Supplementary Fig. S3).

### No gross defect in Sam3^−/−^ mice

**Figure 2:**
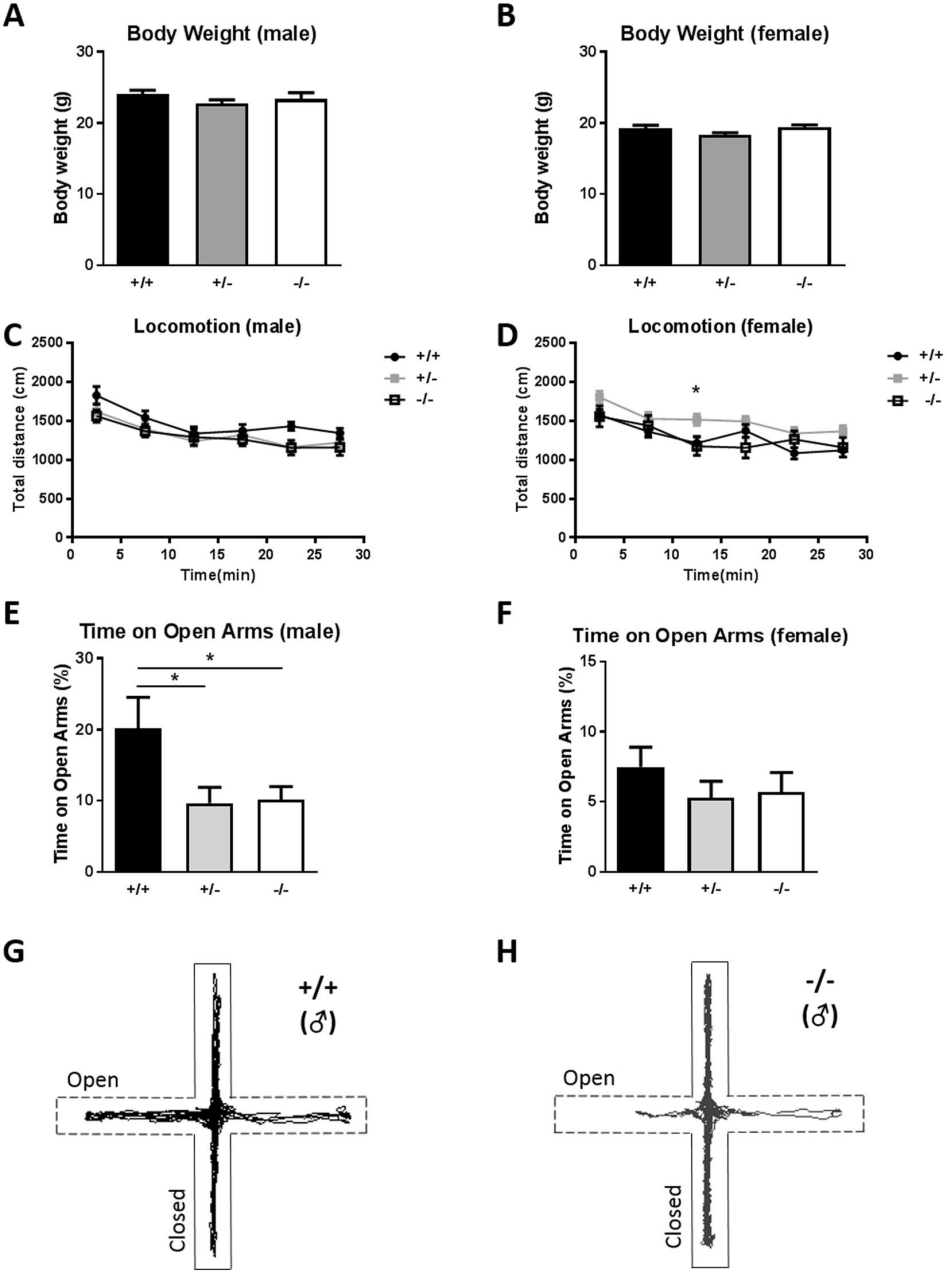
*Sam3* ^−/−^ mice had normal body weight and locomotion; however, male mice showed increased anxiety. (**A**) Body weight of males. *Sam3* ^+/+^, n = 14; *Sam3* ^+/−^, n = 15; *Sam3* ^−/−^, n = 14; one-way ANOVA with Bonferroni post hoc test, F(2,40) = 0.9056, p = 0.4124. (**B**) Body weight of females. *Sam3* ^+/+^, n = 20; *Sam3* ^+/−^, n = 16; *Sam3* ^−/−^, n = 13; one-way ANOVA with Bonferroni post hoc test, F(2,46) = 2.456, p = 0.0969. (**C**) Total distance traveled in the OFT by males. *Sam3* ^+/+^, n = 15; *Sam3* ^+/−^, n = 20; *Sam3* ^−/−^, n = 17; two-way ANOVA with Bonferroni post hoc test, F(2,245) = 1.62, p = 0.2087. (**D**) Total distance traveled in the OFT by females. *Sam3* ^+/+^, n = 19; *Sam3* ^+/−^, n = 19; *Sam3* ^−/−^, n = 13; two-way ANOVA with Bonferroni post hoc test, F(2,240) = 3.26, *p = 0.0470. (**E**) Percentage of time spent in the open arms in the EPM for males. *Sam3* ^+/+^, n = 14; *Sam3* ^+/−^, n = 19; *Sam3* ^−/−^, n = 17; one-way ANOVA with Bonferroni’s post hoc test, F(2,46) = 4.076, *p = 0.0235. (**F**) Percentage of time spent in the open arms in the EPM for females. *Sam3* ^+/+^, n = 20; *Sam3* ^+/−^, n = 19; *Sam3* ^−/−^, n = 13; one-way ANOVA with Bonferroni’s post hoc test, F(2,49) = 0.8294, p = 0.4424. Data are expressed as the mean ± SEM (*p < 0.05). (**G**) Representative video tracking data of male *Sam3* ^+/+^ mice. (**H**) Representative video tracking data of male *Sam3* ^−/−^ mice.

*Sam3* homozygous pups were born at approximately 25%, the expected Mendelian frequency, and showed no visible morphological defects. Prior to various behavior experiments, we initially measured the body weight of the mice at 8 weeks. There was no significant difference in the body weight among the three genotypes, *Sam3* ^+/+^, *Sam3* ^+/−^ and *Sam3* ^−/−^, in males (Fig. 2A; one-way ANOVA with Bonferroni’s post hoc test, F(2,40) = 0.9056, p = 0.4124) and females (Fig. 2B; one-way ANOVA with Bonferroni’s post hoc test, F(2,46) = 2.456, p = 0.0969).

### Increased anxiety and normal locomotion: open field test and elevated plus maze

We initially used the open field test to examine the locomotor activity of the mutants. No significant difference in the locomotor activity was identified between the mutants and the wild types in males (Fig. 2C; two-way ANOVA, F(2,245) = 1.62, p = 0.2087). We did not observe any difference in the locomotor activity in the open field assay among the males of the three genotypes (*Sam3* ^+/+^, *Sam3* ^−/−^, and *Sam3* ^+/−^). In females, however, *Sam3* ^+/−^ showed slightly increased locomotor activity limited to the 10–15 min period of the 30 min test compared to the other two genotypes, *Sam3* ^+/+^ and *Sam3* ^−/−^ (Fig. 2D; two-way ANOVA, F(2,240) = 3.26, *p = 0.0470). Further studies may be required to clarify the biological significance of this finding. We did not identify this discrepancy between *Sam3* ^+/−^ male mice and *Sam3* ^−/−^ male mice. We subsequently used the elevated plus maze to measure the anxiety-like behavior of the mutant mice. The male *Sam3* ^+/−^ and *Sam3* ^−/−^ mice spent significantly less time in the open arms than the wild-type mice (Fig. 2E; one-way ANOVA followed by Bonferroni’s post hoc test, F(2,46) = 4.076, *p = 0.0235), which is also depicted in the representative trace of male mice in the maze (Fig. 2G,H). In contrast, there was no significant difference in the percentage of time that female mice spent in the open arms (Fig. 2F; one-way ANOVA with Bonferroni’s post hoc test, F(2,49) = 0.8294, p = 0.4424) across all genotypes, which indicates a sex difference in this anxiety phenotype. In summary, both male and female *Sam3* ^−/−^ mice exhibited normal locomotion, whereas only male KO mice exhibited increased anxiety.

### Normal learning and memory: fear conditioning

**Figure 3:**
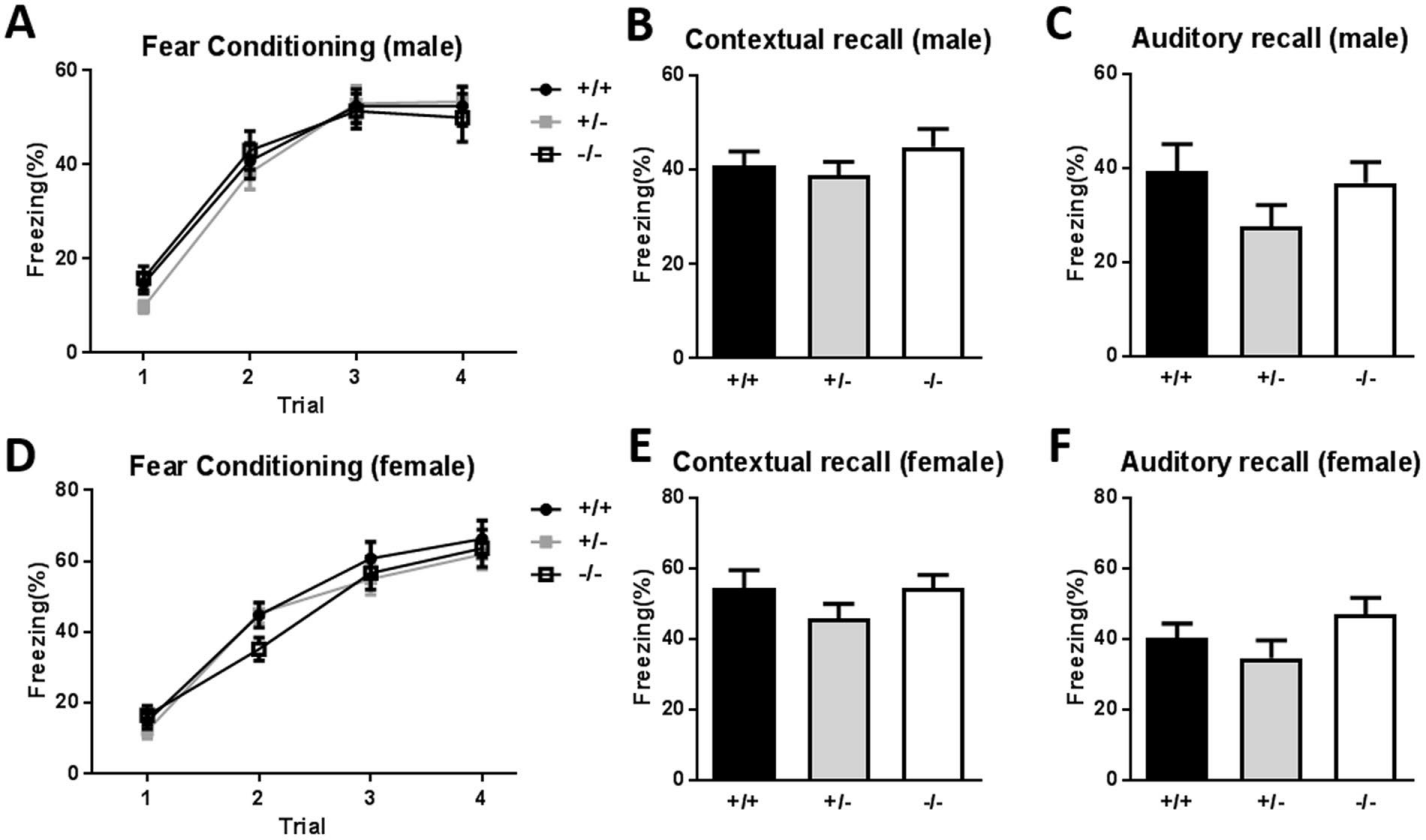
Both male and female *Sam3* ^−/−^ mice showed normal learning and memory. (**A**) Freezing level (%) during 30 sec of tone trials for fear conditioning in male mice. *Sam3* ^+/+^, n = 14; *Sam3* ^+/−^, n = 17; *Sam3* ^−/−^, n = 14; two-way ANOVA with Bonferroni’s post hoc test, F(2,126) = 0.16, p = 0.8563. (**B**) Freezing level (%) during contextual recall in male mice; one-way ANOVA with Bonferroni’s post hoc test, F(2,43) = 0.8970, p = 0.4153. (**C**) Freezing level (%) of tone trial during cued retrieval in male mice; one-way ANOVA with Bonferroni’s post hoc test, F(2,41) = 1.654, p = 0.2038. (**D**) Freezing level (%) during 30 sec of tone trials for fear conditioning in female mice. *Sam3* ^+/+^, n = 17; *Sam3* ^+/−^, n = 12; *Sam3* ^−/−^, n = 13; two-way ANOVA with Bonferroni post hoc test, F(2,117) = 0.54, p = 0.5872. (**E**) Freezing level (%) during contextual recall in female mice; one-way ANOVA with Bonferroni’s post hoc test, F(2,39) = 1.070, p = 0.3528. (**F**) Freezing level (%) of tone trial during cued retrieval in female mice; one-way ANOVA with Bonferroni’s post hoc test, F(2,39) = 1.634, p = 0.2083. Data are expressed as the mean ± SEM.

To characterize the capacity for learning and memory, we assessed fear conditioning followed by contextual or auditory recall. During the training day, the male mice showed similar learning curves in the tone and shock paired conditioning test (Fig. 3A; two-way ANOVA, F(2,126) = 0.16, p = 0.8563). On the retrieval day, the context- or cue-dependent fear memory was tested. There was no significant difference in the freezing response level to the conditioned context or the cue, a tone. All mice elicited normal high-level freezing responses similar to the wild-type mice (Fig. 3B; one-way ANOVA with Bonferroni’s post hoc test, F(2,43) = 0.8970, p = 0.4153, 3 C; one-way ANOVA with Bonferroni’s post hoc test, F(2,41) = 1.654, p = 0.2038). Moreover, the female mutants showed similar levels of freezing during fear conditioning (Fig. 3D; two-way ANOVA, F(2,117) = 0.54, p = 0.5872), contextual retrieval (Fig. 3E; one-way ANOVA, F(2,39) = 1.070, p = 0.3528) and auditory retrieval tests (Fig. 3F; one-way ANOVA with Bonferroni’s post hoc test, F(2,39) = 1.634, p = 0.2083). In summary, both male and female *Sam3*-deficient mice exhibited normal contextual and auditory fear learning and memory.

### Normal sociability and impaired social novelty behavior: three-chamber test

**Figure 4:**
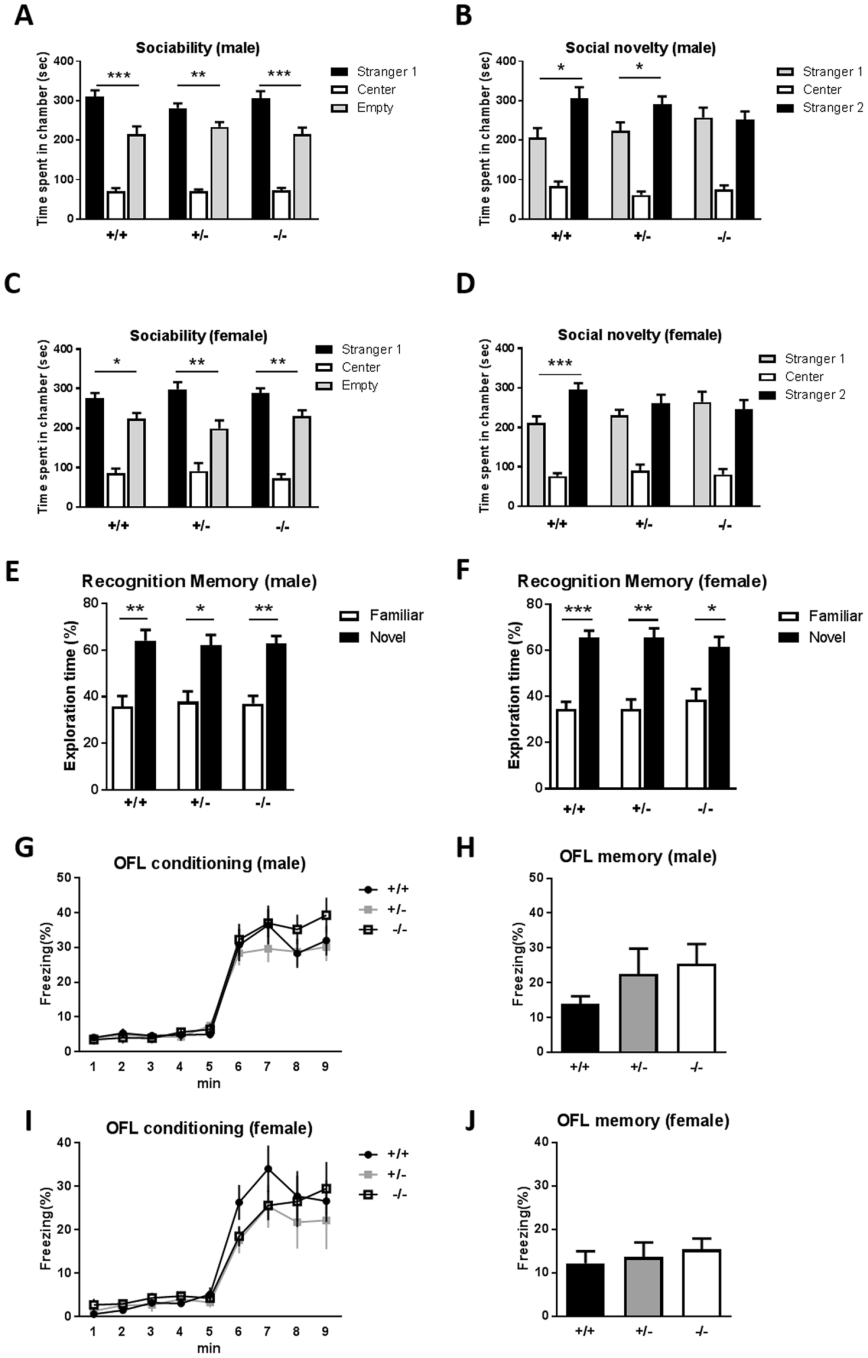
Both male and female *Sam3* ^−/−^ mice showed normal sociability, recognition memory, and empathic fear responses and impaired social novelty preference. (**A**) Time spent in the chamber (sec) during the sociability test for male mice. *Sam3* ^+/+^, n = 11; *Sam3* ^+/−^, n = 18; *Sam3* ^−/−^, n = 17; one-way ANOVA with Bonferroni’s post hoc test, male *Sam3* ^+/+^, F(2,30) = 64.75, ***p = 0.0004; male *Sam3* ^+/−^, F(2,51) = 112.8, **p = 0.0067; male *Sam3* ^−/−^, F(2,48) = 64.80, ***p = 0.0002. (**B**) Time spent in the chamber (sec) during the social novelty test for male mice. One-way ANOVA with Bonferroni’s post hoc test, *Sam3* ^+/+^, F(2,30) = 23.59, *p = 0.0135; *Sam3* ^+/−^, F(2,53) = 46.64, *p = 0.02628; *Sam3* ^−/−^, F(2,48) = 28.03, p > 0.9999. (**C**) Time spent in the chamber (sec) during the sociability test for female mice. *Sam3* ^+/+^, *n* = 15; *Sam3* ^+/−^, *n* = 18; *Sam3* ^−/−^, *n* = 13; One-way ANOVA with Bonferroni’s post hoc test, female *Sam3* ^+/+^, F(2,48) = 55.33, *p = 0.0260; female *Sam3* ^+/−^, F(2,51) = 27.89, **p = 0.0023; female *Sam3* ^−/−^, F(2,36) = 84.07, **p = 0.0048. (**D**) Time spent in the chamber (sec) during the social novelty test for female mice. One-way ANOVA with Bonferroni’s post hoc test, *Sam3* ^+/+^, F(2,48) = 63.10, ***p = 0.0003; *Sam3* ^+/−^, F(2,51) = 27.36, p = 0.7208; *Sam3* ^−/−^, F(2,36) = 22.19,p > 0.9999. (**E**) Exploration time (%) during the novel object recognition test for male mice. *Sam3* ^+/+^, *n* = 14; *Sam3* ^+/−^, *n* = 17; *Sam3* ^−/−^, *n* = 14; two-tailed t-test, F(2,42) = 0.13, *Sam3* ^+/+^, **p = 0.0076; *Sam3* ^+/−^, *p = 0.0150; *Sam3* ^−/−^, *p = 0.0017. (**F**) Exploration time (%) during the novel object recognition test for female mice. *Sam3* ^+/+^, *n* = 20; *Sam3* ^+/−^, *n* = 18; *Sam3* ^−/−^, *n* = 12; two-tailed t-test, F(2,47) = 0.13, *Sam3* ^+/+^, ***p = 0.0001; *Sam3* ^+/−^, **p = 0.0016; *Sam3* ^−/−^, *p = 0.0284. (**G**) Freezing level (%) during OFL conditioning in male mice. *Sam3* ^+/+^, n = 10; *Sam3* ^+/−^, n = 12; *Sam3* ^−/−^, n = 14; two-way ANOVA, F(2,248) = 0.71, p = 0.4990. (**H**) Freezing level (%) during the memory test for observational fear in male mice. One-way ANOVA with Bonferroni’s post hoc test, F(2,33) = 1.162, p = 0.3253. (**I**) Freezing level (%) during the OFL conditioning in female mice. *Sam3* ^+/+^, n = 11; *Sam3* ^+/−^, n = 10; *Sam3* ^−/−^, n = 11; two-way ANOVA, F(2,232) = 0.65, p = 0.5293. (**J**) Freezing level (%) during the memory test for observational fear in female mice. One-way ANOVA with Bonferroni’s post hoc test, F(2,29) = 0.3073, p = 0.7378. Data are expressed as the mean ± SEM (*p < 0.05, **p < 0.01, ***p < 0.001).

Impaired social behavior is one of the major characteristics of autism patients. We used the 3-chamber test to explore the social behavior of *Sam3*-deficient mice. In the sociability test, all three groups of male and female mice spent significantly more time in the chamber with a stranger mouse than with a nonsocial novel object, which indicates normal sociability (Fig. 4A; male *Sam3* ^+/+^, one-way ANOVA with Bonferroni’s post hoc test, F(2,30) = 64.75, ***p = 0.0004; male *Sam3* ^+/−^, F(2,51) = 112.8, **p = 0.0067; male *Sam3* ^−/−^, F(2,48) = 64.80, ***p = 0.0002; 4 C, female *Sam3* ^+/+^, F(2,48) = 55.33, *p = 0.02601; female *Sam3* ^+/−^, F(2,51) = 27.89, **p = 0.0023; female *Sam3* ^−/−^, F(2,36) = 84.07, **p = 0.0048). However, in the social novelty test, the male *Sam3* ^−/−^ mice showed no preference for stranger 2 over stranger 1, whereas the *Sam3* ^+/+^ and *Sam3* ^+/−^ male mice spent more time in the novel mouse chamber (stranger 2) than in the familiar mouse chamber (stranger 1) (Fig. 4B; one-way ANOVA with Bonferroni’s post hoc test, *Sam3* ^+/+^, F(2,30) = 23.59, *p = 0.0135; *Sam3* ^+/−^, F(2,53) = 46.64, *p = 0.0263; *Sam3* ^−/−^, F(2,48) = 28.03, p > 0.9999). The female *Sam3* ^+/−^ and *Sam3* ^−/−^ mice did not show a preference for stranger 2 over stranger 1, whereas the *Sam3* ^+/+^ females spent more time in the chamber with stranger 2 than in the chamber with stranger 1 (Fig. 4D; one-way ANOVA with Bonferroni’s post hoc test, *Sam3* ^+/+^, F(2,48) = 63.10, ***p = 0.0003; *Sam3* ^+/−^, F(2,51) = 27.36, p = 0.7208; *Sam3* ^−/−^, F(2,36) = 22.19, p > 0.9999). In summary, both male and female *Sam3*-deficient mice exhibited impaired social novelty preference or social memory.

### Normal novelty recognition and memory: novel object recognition test

To ensure that the social novelty deficit was not a result of a lack of novelty recognition, we tested the novel object recognition assay. After 10 min of the familiarization phase, all groups of male mice showed a significant preference for the novel object in the test (Fig. 4E; two-tailed t-test, *Sam3* ^+/+^, **p = 0.0076; *Sam3* ^+/−^, *p = 0.0150; *Sam3* ^−/−^, **p = 0.0017). Similar to males, all groups of female mice showed normal recognition memory (Fig. 4F; two-tailed t-test, *Sam3* ^+/+^, ***p = 0.0001; *Sam3* ^+/−^, **p = 0.0016; *Sam3* ^−/−^, *p = 0.0284). These results suggest that the impairment of the mutants in the social novelty test was not a result of a lack of novelty recognition.

### Normal empathy: observational fear learning

Individuals with autism often display deficits in empathy and social skills^19^. Observational fear learning (OFL) behavior is a rodent model of emphatic responses. To examine the empathic fear response of *Sam3* KO mice, we measured the freezing level of the observer mice, while the demonstrator mice received repetitive foot shocks. There was no significant difference among the *Sam3* ^+/+^, *Sam3* ^+/−^ and *Sam3* ^−/−^ male mice in the OFL conditioning (Fig. 4G; two-way ANOVA, F(2,248) = 0.71, p = 0.4990). In the retrieval test, there was no significant difference among the genotypes (Fig. 4H; one-way ANOVA, F(2,33) = 1.162, p = 0.3253). Similarly, the *Sam3* ^+/+^, *Sam3* ^+/−^ and *Sam3* ^−/−^ female mice did not exhibit a significant difference in the OFL conditioning (Fig. 4I; two-way ANOVA, F(2,232) = 0.65, p = 0.5293) or retrieval (Fig. 4J; one-way ANOVA, F(2,29) = 0.3073, p = 0.7378). In summary, both male and female mice showed normal empathy.

### Impaired social communication: scent marking

**Figure 5:**
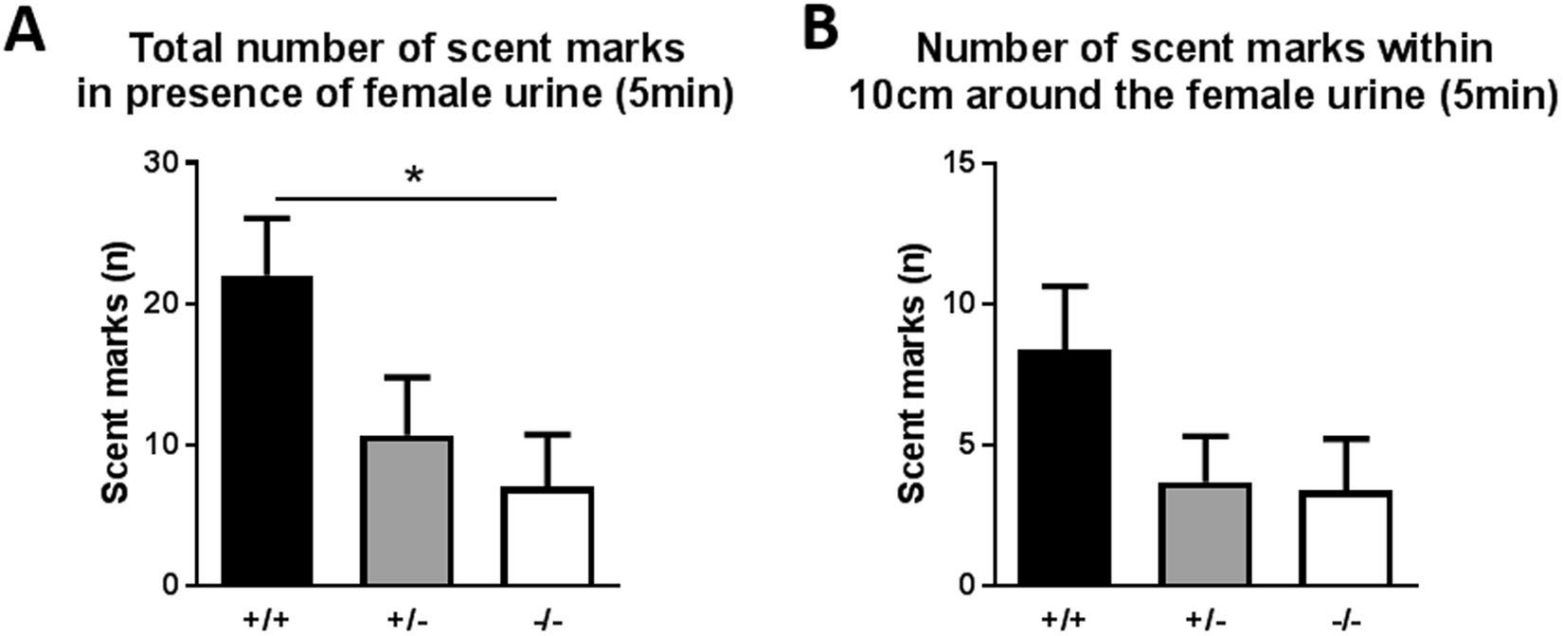
*Sam3* ^−/−^ male mice showed impaired social communication. (**A**) Total number of scent marks (n) in the presence of female urine. *Sam3* ^+/+^, n = 10; *Sam3* ^+/−^, n = 10; *Sam3* ^−/−^, n = 13; one-way ANOVA with Bonferroni’s post hoc test, F(2,30) = 3.846, p = 0.0326. (**B**) Number of scent marks (n) within 10 cm around the female urine. One-way ANOVA with Bonferroni’s post hoc test, F(2,30) = 2.017, p = 0.1507. Data are expressed as the mean ± SEM (*p < 0.05).

Mice communicate via olfactory^20–22^ and acoustic signals^23,24^. Based on several reports regarding the low level of vocalization in C57BL/6N^25,26^ mice, the genetic background of the mutation, we focused on scent marking behavior in *Sam3* KO mice instead of ultrasonic vocalization. A female urine-elicited scent marking is a type of chemical communication in social groups^21,22^. Because we utilized female urine as the scent, we tested only male mice for this specific behavior. During the 5-min test session, the total number of scent marks was counted. As shown in Fig. 5A, the male *Sam3* ^−/−^ mice made fewer scent marks than the *Sam3* ^+/+^ and *Sam3* ^+/−^ mice in the open field box (one-way ANOVA followed by Bonferroni’s post hoc test, F(2,30) = 3.846, *p = 0.0326). This result indicated that *Sam3* ^−/−^ mice showed reduced social communication. The number of male urine marks 10 cm around the female urine tended to decrease in both the *Sam3* ^+/−^ and *Sam3* ^−/−^ mice; however, the data were not statistically significant (Fig. 5B; one-way ANOVA followed by Bonferroni’s post hoc test, F(2,30) = 2.017, p = 0.1507). Overall, the total number of scent marks was significantly reduced in the *Sam3* ^−/−^ male mice.

### Increased repetitive behaviors: marble burying test and self-grooming

**Figure 6:**
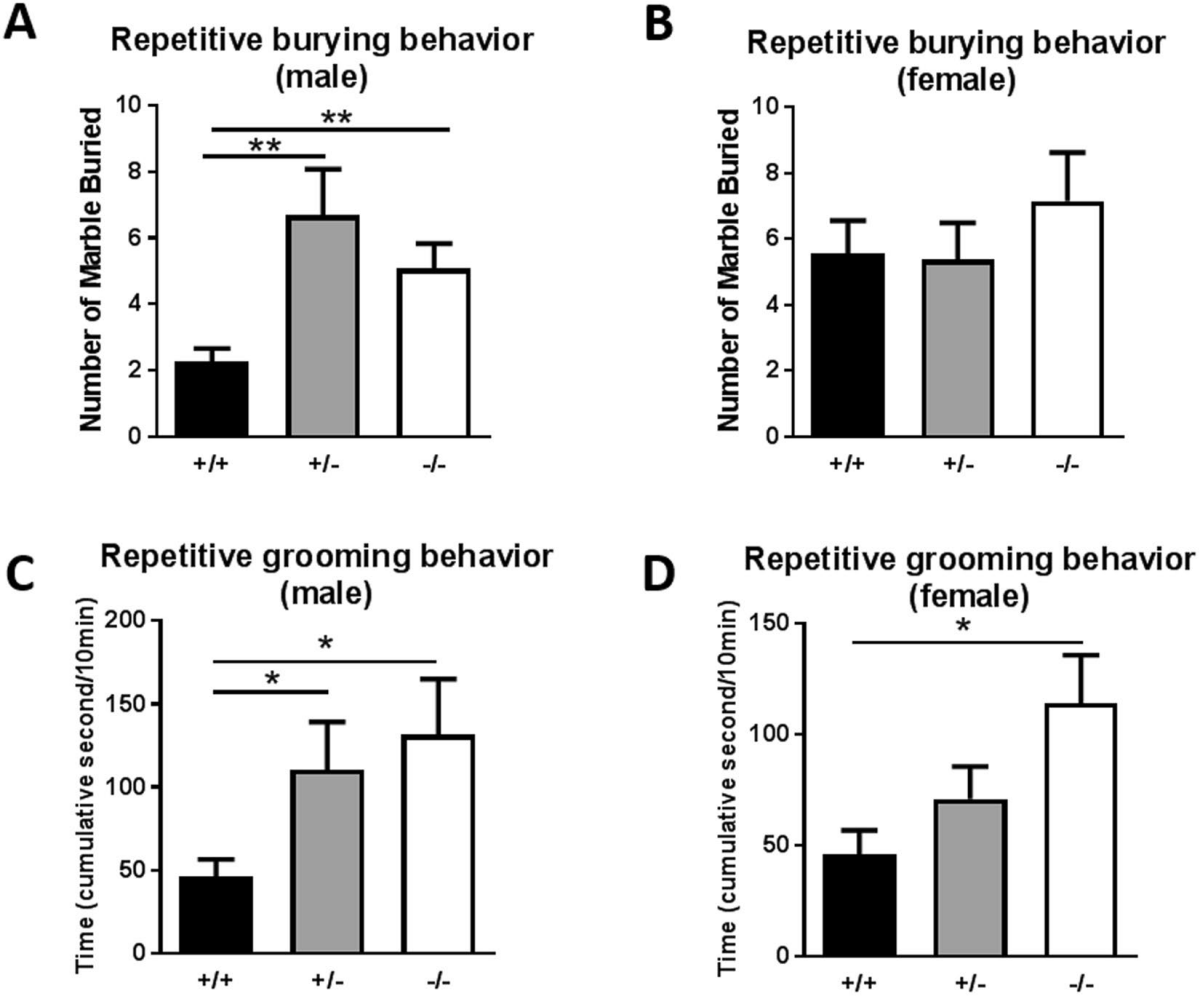
Increased repetitive behaviors were identified for *Sam3* ^−/−^ and *Sam3* ^+/−^ male mice and *Sam3* ^−/−^ female mice. (**A**) Number of marbles buried during the marble burying test by male mice. *Sam3* ^+/+^, n = 15; *Sam3* ^+/−^, n = 19; *Sam3* ^−/−^, n = 13; one-way ANOVA with Bonferroni’s post hoc test, F(2,44) = 4.621, *p = 0.0151. (**B**) Number of marbles buried during the marble burying test by female mice. *Sam3* ^+/+^, n = 19; *Sam3* ^+/−^, n = 17; *Sam3* ^−/−^, n = 13; one-way ANOVA with Bonferroni’s post hoc test, F(2,49) = 0.5950, p = 0.5555. (**C**) Cumulative time spent self-grooming in male mice. *Sam3* ^+/+^, n = 12; *Sam3* ^+/−^, n = 11; *Sam3* ^−/−^, n = 10; one-way ANOVA with Bonferroni’s post hoc test, F(2,30) = 3.846, *p = 0.0526. (**D**) Cumulative time spent self-grooming in female mice. *Sam3* ^+/+^, n = 10; *Sam3* ^+/−^, n = 10; *Sam3* ^−/−^, n = 11; one-way ANOVA with Bonferroni’s post hoc test, F(2,28) = 4.318, p = 0.0232. Data are expressed as the mean ± SEM (*p < 0.05, **p < 0.01). Data are expressed as the mean ± SEM.

To investigate whether mutant mice show repetitive behaviors, we analyzed the performance of mice in the marble burying test and self-grooming test. In the marble burying test, the male *Sam3* ^+/−^ and *Sam3* ^−/−^ mice buried significantly more marbles than the *Sam3* ^+/+^ mice (Fig. 6A; one-way ANOVA followed by Bonferroni’s post hoc test, F(2,44) = 4.621, *p = 0.0151). For the females, there was no significant difference in this behavior among the three groups of mice (Fig. 6B; one-way ANOVA followed by Bonferroni’s post hoc test, F(2,49) = 0.5950, p = 0.5555).

Repetitive behavior was subsequently evaluated by scoring the time the mice spent grooming during 10 min after a habituation session. Consistent with the marble burying result, the *Sam3* mutant mice exhibited unusually high spontaneous repetitive grooming behavior. In the self-grooming test, the male *Sam3* ^+/−^ and *Sam3* ^−/−^ mice spent a significantly longer time grooming than the *Sam3* ^+/+^ mice (Fig. 6C; one-way ANOVA followed by Bonferroni’s post hoc test, F(2,30) = 3.846, *p = 0.0523). For females, the *Sam3* ^−/−^ mice also displayed a longer duration of self-grooming than the *Sam3* ^+/−^ and *Sam3* ^+/+^ mice (Fig. 6D; one-way ANOVA followed by Bonferroni’s post hoc test, F(2,28) = 4.318, *p = 0.0232). Although only the male *Sam3* KO showed a significant increase in marble burying as previously described, both the male and female *Sam3* KO mice showed increased levels of self-grooming behavior compared to the wild-type mice.

## Discussion

In this study, we first generated *Sam3* KO mice and assessed behavioral phenotypes that are relevant to the symptoms of human ASDs. Our data clearly showed that *Sam3*-deficient mice exhibited behavioral impairments in social interactions and social communication and increased repetitive behaviors, which are the core symptoms required for an ASD diagnosis in humans.

In our study, *Sam3* KO mice showed increased anxiety as well as impairments in the three core domains of ASD (Fig. 2E). Autistic children exhibit various symptoms in addition to the three core symptoms^27–29^. Gene-based association studies have identified many genes associated with ASD. However, there are very few genetic mouse models that meet the criteria for the three core symptoms of autism. Three representative mouse models have been reported to date that meet the criteria: Cntnap2 KO mice, ProSAP1/shank2 KO mice and Shank3A KO mice^30,31,32^. Although these three genetic mouse models show reduced social interaction, reduced social communication and increased repetitive behaviors, they also present unique impairments in other behaviors. For example, Cntnap2 KO mice exhibit hyperactivity and epileptic seizures^31,33^. ProSAP1/shank2 KO mice show hyperactivity and impaired spatial learning and memory^30,34^. Shank3A KO mice show impaired learning and memory as well as motor coordination^32,35^. Shank3 KO mice with a non-sense mutation at arginine 1117 (R1117X) also show impaired prepulse inhibition^36^. Thus, the development of additional mouse models with these various symptoms is necessary to understand individual dysfunctions.

Interestingly, *Sam3* KO did not affect sociability; however, it selectively impaired social novelty preference. Sociability is one of the most direct phenotypes used to measure social interaction; however, accumulating evidence from human patients suggests that an impaired social novelty preference is also a critical parameter of ASD. As previously reported, some individuals with ASD are unable to differentiate between familiar and unfamiliar acquaintances because of their difficulty in processing facial configurations^37,38^. fMRI studies have indicated that individuals with ASD showed a lack of fusiform gyrus activation when viewing unfamiliar faces. The fusiform gyrus is involved in discriminating between familiar and unfamiliar faces. Another study showed that children with ASD had significantly higher levels of cortisol, the primary stress hormone, in response to novel social interactions than typical peer control children, which resulted in an inappropriate approach or reaction to unfamiliar peers^39^. Difficulties in facial discrimination or feelings of stress during novel or unfamiliar social situations affect the social interactions of ASD patients. There are several mouse models that show a lack of social novelty preference but not sociability: integrin β3 KO mice^40^, Shank 3B KO mice^35^, ProSAP1/Shank2 KO mice^34^ and mice with oxytocin receptor (OXTR) knockdown in the lateral septum^41^. However, whether *Sam3* and these related genes share common molecular pathways or brain circuits regarding the mechanism of selectively impaired social novelty preference but not impaired sociability remain unclear. We confirmed that the impairment in the social novelty test is not a result of a lack of novel object recognition memory (Fig. 4B,D).

In humans, the prevalence of ASD is 4-fold higher in boys than in girls, which supports the existence of gender differences in ASD development^42,43,44^. More interestingly, several previous studies have suggested that boys with ASD present more repetitive and restricted behavior than girls with ASD^45,46^. The marble burying test and the self-grooming test are well-established models used to measure repetitive behaviors in rodents. In our study, we identified impairments in both behaviors only in *Sam3* KO male mice (Fig. 6A,C), whereas female *Sam3* KO mice exhibited normal burying behavior in the marble burying test (Fig. 6B). The low number of buried marbles of WT males was a result of the type of bedding, which included a thick and heavy type of bedding selected to maximize the difference between WT and MT mice (Fig. 6A). Initially, with light bedding, WT male mice buried an average of 15 marbles (data not shown). Thus, it was difficult to observe the difference from the male MT mice. In this condition, we identified increased repetitive behaviors only in male KO mice. Another issue that occurred was that female wild-type mice (5.55 ± 1.014) (Fig. 6B) buried more marbles than male wild-type mice (2.267±0.4079) (Fig. 6A). This difference has also been reported in other studies^47,48^; however, the gender difference in repetitive behaviors remains unclear. For self-grooming, although the female KO group showed increased self-grooming, the female heterozygous (Het) group did not exhibit increased self-grooming (Fig. 6D). Considering the haploinsufficiency of risk genes related to autism in patients, the significant increase in self-grooming only in the male Het group suggests the possibility that *Sam3* gene dysfunction may be associated with gender differences in autism patients. In addition to the effects of gender on repetitive behaviors, we also identified increased anxiety only in male *Sam3* HET and KO mice (Fig. 2E). Recent studies have demonstrated that anxious children with ASD exhibited more repetitive behaviors than non-anxious children^7,48,49^. Given that SAM3 expression is high in the medial habenular nucleus (MHN)^15^, which is a thoroughly investigated brain region in anxiety disorders, it is possible that the *Sam3* gene deletion in the MHN may be associated with the increased anxiety. Regardless of the assumption that the *Sam3* deletion in the MHN is critical for an anxiogenic phenotype, it remains unclear how the sex difference in anxiety occurs and how anxiety and repetitive behaviors are associated in *Sam3* KO male mice. Although research on sex-bias in ASD appears to be increasing, there is limited knowledge regarding the underlying mechanisms. Thus, understanding the underlying mechanisms of *Sam3* deficiency may provide clues to answer questions regarding sex differences in ASD as well as ASD pathophysiology.

Chemokines, which are immune-related proteins, have been implicated in ASDs^11,51,52^. In humans, several types of plasma chemokines, including MIP-1 alpha, have been reported to be significantly lower in ASD children than their siblings without ASD^13,53^. A sequence homology study indicated that the SAM3 protein is distantly related to MIP-1 alpha, the CC chemokine family^15,16^. A recent study has suggested that *Sam3* may also play a role in middle cerebral artery occlusion (MCAO)-mediated immune responses by maintaining M2 polarization of microglia^54^. M2 polarized microglia express cytokines and receptors that inhibit inflammation and subsequently exert beneficial roles in homeostasis and cell survival. Therefore, one potential explanation for the behavioral abnormalities identified in *Sam3* KO mice is that a lack of *Sam3* release may lead to the loss of the homeostatic control of inflammation, which may ultimately result in the abnormal brain development or function present in ASD patients^51^. Another potential underlying mechanism is that *Sam3* may directly act on neurons. As previously discussed, a deletion of Cntnap2, Shank2 or Shank3, respectively, has been reported to induce three core phenotypes of ASD. Whether these molecules and SAM3 are directly associated is unknown; however, a better understanding of the general roles of each gene in the brain is required. Considering the role of Cntnap2 in the neuronal migration of cortical projection neurons, alterations in connectivity across diverse brain regions may be important for ASD development, and its specific expression in the brain may be directly associated with extra phenotypes, such as hyperactivity and defects in spatial learning and memory. Shank2 and Shank3 are multidomain scaffolding proteins that anchor NMDA, AMPA and metabotropic glutamate receptors in the postsynaptic membrane, assemble signaling molecules and G-protein coupled receptors, and regulate calcium homeostasis and synaptic plasticity^30,32^. Defects in synaptic transmission and synaptic plasticity are well-accepted molecular and cellular mechanisms for ASD pathophysiology. Importantly, accumulating evidence has indicated that chemokines couple with a subset of glutamate receptors and modulate synaptic activity^27,42^. Future studies are required to ascertain the specific cell types that express SAM3 and identify its specific receptors to elucidate the underlying cellular and molecular mechanisms of *Sam3* deficiency-mediated ASD phenotypes.

In conclusion, we report experimental results that show a deficiency in the brain-specific chemokine *Sam3* results in the development of ASDs in mice and suggest that *Sam3*, a soluble factor with a preventive function in ASD pathogenesis, may comprise a target for ASD treatment.

## Materials and Methods

### Animals

The C57BL6/NTac strain was used for all experiments. Mice were provided with free access to food and water under a 12 hr light/dark cycle with the light cycle beginning at 8:00 am. The mice were 8–17 weeks of age at the time of behavioral testing, which was performed during the light phase. All animal experiments were performed in accordance with a protocol approved by the Institutional Animal Care and Use Committee (IACUC) of Korea Advanced Institute of Science and Technology (KAIST -site of the experiment) and the Institute for Basic Science (IBS), Korea. All methods were performed in accordance with relevant guidelines and regulations.

### Generation of targeted mouse *Sam3* KO mutant and genotyping

The TALEN vectors that targeted exon 4 of *Sam3* were designed and constructed by ToolGen (Seoul, Korea). The synthesis and microinjection of TALEN mRNAs into the cytoplasm of fertilized eggs obtained from C57BL/6NTac breeding females were performed as previously described^55^. TALEN-mediated *Sam3* F0 mice were screened via T7E1 assay as previously described^56^. For the assay, genomic DNA was prepared from the tail and amplified using TALEN target site primers. The primers used for genotyping included mouse SAM3 forward (5′-GCATAGAGAAGGGGCTGA-3′) and mouse SAM3 reverse (5′- GAGGAGTCACATCTGCAG-3′). The founder line of a *Sam3* heterozygous mouse (F0) was crossed to and maintained in the C57BL/6NTac background. Heterozygous breeding pairs were used to generate *Sam3* homozygous mice (*Sam3* ^−/−^) and wild-type littermates.

### RNA preparation and reverse transcription PCR (RT-PCR)

Total RNAs were extracted from whole brain tissues of adult *Sam3* ^−/−^, *Sam3* ^+/−^, and wild-type littermate mice using GeneAll Hybrid-R (GeneAll Biotechnology, Korea). cDNA was synthesized following the manufacturer’s protocols (SuperScript IV VILO Master Mix, Invitrogen). Two sets of primers were used: a forward primer (5′-ATG GAG AGG CCC ACC AG-3′) and a reverse primer (5′-CAG GAG CAT TTG ACC GTC TG-3′) to determine the truncated mRNA and a forward primer (5′-TGG CTG TGG ACG TGT CCG-3′) and a reverse primer (5′-TTA CCG TGT GAC CTT GGT G-3′) to confirm the absence of the deletion site. The *glyceraldehyde-3-phosphate dehydrogenase (GAPDH)* gene was used as an internal control as previously described^57^.

### Behavioral tests

Prior to the behavioral tests, all mice were placed in the behavior room for 1 hr for room habituation with white noise (65 dB). Every behavior test was performed in a sound-proof chamber with white noise (65 dB) and dim light (10 lux), except for the elevated plus maze (5 lux). Between each test session, the chambers and objects were cleaned with 70% ethanol. After the test, all mice were returned to their home cages.

### Open field test (OFT)

The OFT was conducted as previously reported^58^. Briefly, each mouse was placed in a white acryl chamber (40 × 40 × 40 cm) for 30 mins to measure its exploratory activity. At the beginning of the test, the mouse was placed in the corner of the arena. Spontaneous movement was video recorded and automatically analyzed with EthoVision XT software, version 9 (Noldus, Wageningen, Netherlands).

### Elevated plus maze (EPM) test

The EPM was performed as previously described^58^, with minor modifications. Briefly, each mouse was placed in the elevated plus maze and allowed to freely move within the maze for 5 mins. The mouse was first placed in the center (8 × 8 cm) facing the open arms. The light intensity in the chamber was 5 lux. The amount of time spent in each arm was video recorded and automatically analyzed using EthoVision XT software. The time spent in the open arms was reported as the percentage of time spent in the open arms (time in open arms/total time (open arm + closed arm) × 100).

### Fear conditioning, contextual and auditory recall

Conventional Pavlovian fear conditioning was performed as previously described^58^, with minor modifications. One day before the conditioning day, each mouse was habituated in a cued recall box for 20 min. On the conditioning day, the mouse was placed in the conditioning chamber (Coulbourn Instruments). After 3 min of exploration, a 30-sec (86 dB, 3000 Hz) auditory conditioned stimulus (CS) was delivered. In the last 2 sec of the CS, an aversive unconditioned stimulus (US, 1 sec foot shock at 0.7 mA) was delivered. For the conditioning, the mice underwent four CS-US pairs separated by 150-sec intervals. Twenty-four hours after training, the contextual fear memory was tested in the same chamber for 5 min in the absence of the auditory stimulus and shock. After 3 hrs, the auditory fear memory was tested in a cued recall box, which differed from the conditioning chamber. After 10 min of exploration time, the 30-sec auditory CS was delivered. The freezing behavior of the mouse was recorded and automatically analyzed with FreezeFrame software (Actimetrics) using the significant motion pixels (SMP) algorithm.

### Three-chamber test

The three-chamber test was conducted as previously described^58^, with minor modifications. The subject mouse was habituated to the empty chamber for 10 min before the sociability session. In the sociability session, the subject mouse was allowed to explore three chambers (object, center, and stranger 1) for 10 min. The chamber location for the object and stranger 1 was randomly assigned for each animal. In the social novelty session, the subject mouse was allowed to explore three chambers (stranger 2, center, and stranger 1) for an additional 10 min. In this session, a novel, unfamiliar, stranger 2 mouse was placed in the empty cylinder in the sociability session. The amount of time spent in each chamber was video recorded and automatically analyzed using EthoVision XT software, version 9 (Noldus, Wageningen, Netherlands).

### Novel object recognition test

The novel object recognition test was administered using previously described methods^59^, with minor modifications. Each mouse was placed in the chamber used in the open field test. Because the mouse had experienced the chamber in previous tests, the habituation session was omitted. In the familiarization session, two objects (small soccer balls, 7 cm in diameter) were placed 5 cm away from the walls in a symmetric position from the center of the chamber. During 10 min, the mouse freely explored two identical objects. Between the familiarization session and test session, there was a 10 min intersession interval (ISI). During the ISI, one object was randomly replaced with a novel object (black pyramid, 8 × 7.5 × 6 cm) in the same location. During the test session, the mouse was allowed to freely explore the two objects (familiar vs novel objects) for 10 min. The object exploration time was automatically measured and analyzed using EthoVision XT software, version 9 (Noldus, Wageningen, Netherlands).

### OFL test

The OFL test was performed as previously described^60^. Each mouse was placed in a modified passive avoidance cage (Coulbourn Instruments, Whitehall, PA, USA) for OFL conditioning and retrieval. The freezing behavior of the observer mouse was recorded and automatically analyzed with FreezeFrame software (Actimetrics).

### Scent marking test

The scent marking test was conducted as previously reported^61,62^, with minor modifications. Before the test, urine was collected from C57BL6/NTac females on the day of the experiment. Clean paper (Strathmore Drawing Paper Premium, 400 series; Strathmore Artist Papers, Neenah, WI, USA) in the chamber (40 × 40 × 40 cm) was used for the open field test, and 15 µl fresh female urine were pipetted onto the center of the paper. In the test session, a male mouse freely explored the chamber with the female urine. After the 5-min test, the mouse was returned to its home cage. The marked sheets of Strathmore paper were treated with ninhydrin spray (LC-NIN-16; TritechForensics Inc., Southport, NC, USA) and dried for 24 hrs, which allowed visualization of the urine marks as purple spots. To analyze the urine marks, the number of scent marks was measured by placing a transparent grid (each 1 × 1 cm) over the dried substrate paper. The total number of grids that contained scent marks was counted for each genotype. The number of scent marks within an area of 10 × 10 cm around the female urine spot was also counted. This analysis was confirmed by a second experimenter.

### Marble burying test

The test was performed as previously described^63^. Each mouse was placed in the center of a cage (20 × 30 × 15 cm) with 3-cm deep clean bedding. Twenty navy glass marbles (14 mm diameter) were gently arranged in an equidistant 4 × 5 array on top of the bedding. Following the 30-min testing session, the marbles covered with bedding more than two-thirds deep were counted as buried marbles. This analysis was confirmed by a second experimenter.

### Self-grooming test

The self-grooming test was performed as described^64,65^. Briefly, an individual mouse was placed in a clean, transparent cage (20 × 30 × 15 cm) with no bedding. Each mouse was allowed a 10-min habituation session, and self-grooming was subsequently measured for an additional 10 min to assess the cumulative time spent grooming all body regions. The experimenter sat at a distance approximately 2 m from the test cage and recorded the cumulative time spent spontaneously grooming using a stopwatch program. The test was video recorded using EthoVision XT software, version 9 (Noldus, Wageningen, Netherlands), and the analysis was confirmed by a second experimenter.

### Statistical analysis

Data were analyzed using GraphPad Prism 7.03 (GraphPad Software Inc., California). We performed one-way ANOVA with Bonferroni’s multiple comparisons *post hoc* test to evaluate the differences among the three groups. We also performed two-way ANOVA (Group × Time interaction) with Bonferroni’s multiple comparisons *post hoc* test. All data are presented as the mean ± SEM.

### Availability of materials and data

All materials and data in this manuscript are available to Editorial Board Members and referees.

## Electronic supplementary material

Supplementary Info
