# Supplementary material for: Deficiency of a brain-specific chemokine-like molecule, SAM3, induces cardinal phenotypes of autism spectrum disorders in mice
Source: Sci Rep. 2017 Nov 28;7:16503. doi: 10.1038/s41598-017-16769-5 (PMC5705707; doi:10.1038/s41598-017-16769-5)
Supplement: Supplementary file 1 — Supplementary Info [file 41598_2017_16769_MOESM1_ESM.pdf]

# Deficiency of a brain-specific chemokine-like molecule, SAM3, induces cardinal phenotypes of autism spectrum disorders in mice

**Sujin Kim[1, 2], Boyoung Lee[1], Jung-Hwa Choi[3], Jong-Hyun Kim[1, 4, 5],**

**Cheol-Hee Kim[3*], and Hee-Sup Shin[1, 2*]**

[1] Center for Cognition and Sociality, Institute for Basic Science, Yuseong-gu, Daejeon 34141, Republic of Korea;

[2] Basic Science, IBS School, University of Science and Technology, Daejeon 34113, Republic of Korea;

[3] Department of Biology, Chungnam National University, Daejeon 34134, Republic of Korea;

[4] Center for Functional Connectomics, Brain Science Institute, Korea Institute of Science and Technology, Seoul 02797, Republic of Korea;

[5] Laboratory of Cell Death and Human Diseases, Department of Life Sciences, School of Life Sciences, Korea University, Seoul 02841, Republic of Korea.

*Cheol-Hee Kim: zebrakim@cnu.ac.kr

*Hee-Sup Shin: shin@ibs.re.kr

# Supplementary Method

### RNA preparation and reverse transcription PCR (RT-PCR)

Total RNAs were extracted from whole brain tissues of adult *Sam3*$^{-/-}$, *Sam3*$^{+/-}$, and wild-type littermate mice using GeneAll Hybrid-R (GeneAll Biotechnology, Korea). cDNA was synthesized following the manufacturer's protocols (SuperScript IV VILO Master Mix, Invitrogen). One sets of primers were used: a forward primer (5'-TGG CTG TGG ACG TGT CCG-3') and a reverse primer (5'-TTA CCG TGT GAC CTT GGT G-3') to confirm the absence of the deletion site. The *glyceraldehyde-3-phosphate dehydrogenase (GAPDH)* gene was used as an internal control as previously described[1].

### Quantitative real time PCR (qRT-PCR)

cDNA was prepared as described in RT-PCR. The obtained cDNA was subjected to the RT-qPCR protocol of qRT-PCR was performed using-2X SensiFAST Probe H-ROX mix (Bioline) using TaqMan Gene Expression assays (Integrated DNA Technologies, USA) and Bio-Rad CFX96 Real-time System (Bio-Rad, USA). The comparative $C_T$ method was used for analysis. All the value was normalized to average of Sam3+/+ control samples. The following primers was sued. Sam1; forward primer (5'-TCT TTC CAA TGA GCA CTA GGA G-3'), reverse primer (5'-GCA CAA GCA CTT ATC CAC AAG-3'), probe (5'-/6-FAM/CCA TTG CCA/ZEN/TTC TCT GAA AAA TCC AGC C/IABkFQ/-3'); Sam2; forward primer (5'-GAT GCA GGA GCG GTG AG-3'), reverse primer (5'-TTT CCT TGT GTT GCT TTC TGC-3'), probe (5'-/6-FAM/TAG CGA TGG/ZEN/TCC GGT AGC TTC C/IABkFQ/-3'); Sam3; forward primer (5'-GAG TCT AGA TGC CAA GCT CAG-3'), reverse primer (5'-TCA GAA TGG AGA GAG ACC AGA-3'), probe (5'-/6-FAM/CAG CTT CCT/ZEN/CTT AGC CCC TCC AG/IABkFQ/-3'); Sam4; forward primer (5'-TCG GCT GCT CTT TCT AGT CTA-3'), reverse primer (5'-CGT GAA TGT CCC TTT GTA ACT G-3'), probe (5'-/6-FAM/CAA AAC TTC/ZEN/TTG GGC TTG GAG GAC C/3IABkFQ/-3'); Sam5; forward primer (5'-GCT GTG ACT TGT TAA TCA ACC G-3'), reverse primer (5'-GTA GAA TTT CCA GGG AGC CA-3'), probe (5'/6-FAM/CGA TTG TCA/ZEN/GGA GAC CGT GGT GG/IABkFQ/-3'); GAPDH; forward primer (5'-AAT GGT GAA GGT CGG TGT G-3'), reverse primer (5'-GTG GAG TCA TAC TGG AAC ATG TAG-3'), probe (5'-/6-FAM/TGC AAA TGG/ZEN/CAG CCC TGG TG/IABkFQ/-3').

### Statistical analysis

Data were analyzed using GraphPad Prism 7.03 (GraphPad Software Inc., California). We performed one-way ANOVA with Bonferroni's multiple comparisons *post hoc* test to evaluate the differences among the three groups. We also performed two-way ANOVA (Group × Time interaction) with Bonferroni's multiple comparisons *post hoc* test. All data are presented as the mean±SEM.

## References

1. Lee, S. E. *et al.* Rebound burst firing in the reticular thalamus is not essential for pharmacological absence seizures in mice. *Proc. Natl. Acad. Sci.* **111,** 11828–11833 (2014).

## Supplementary Figures

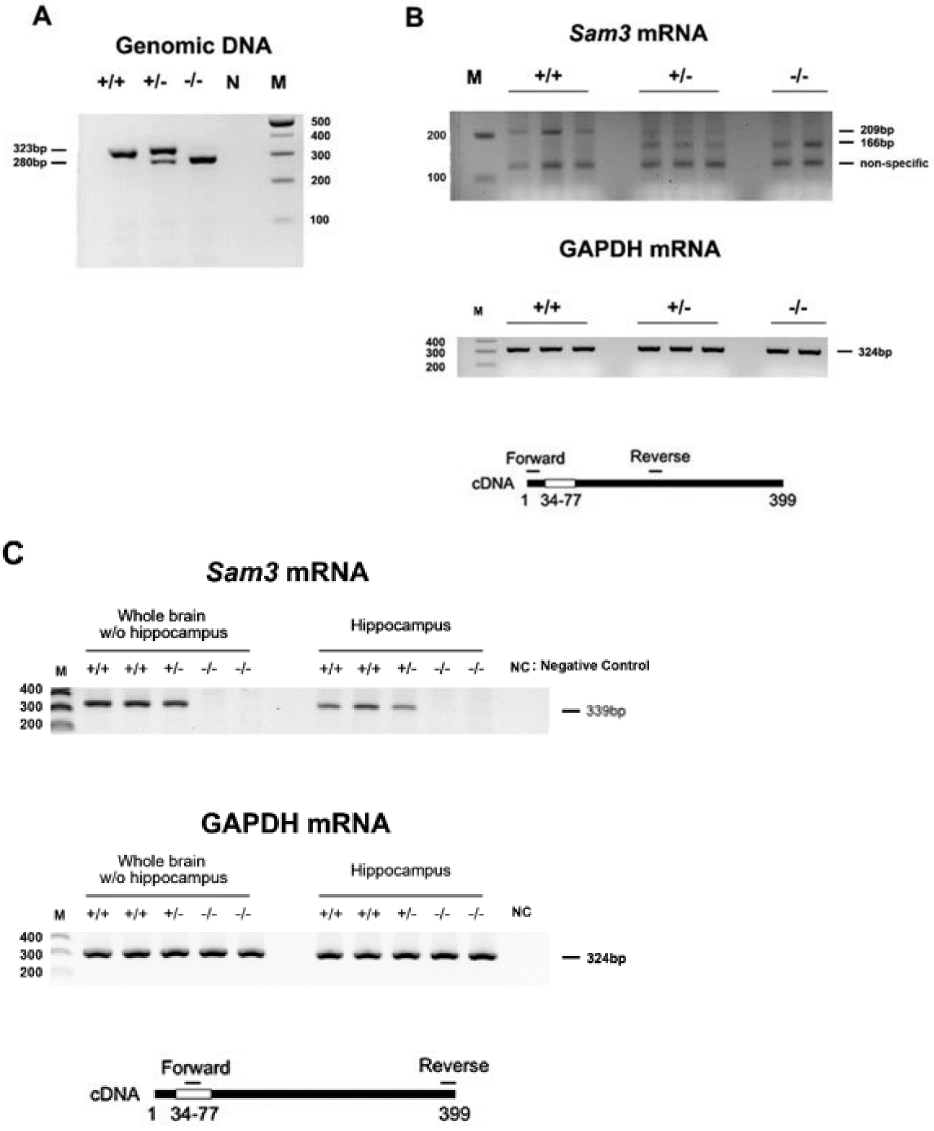


**Supple Fig. 1 Full length blots.**

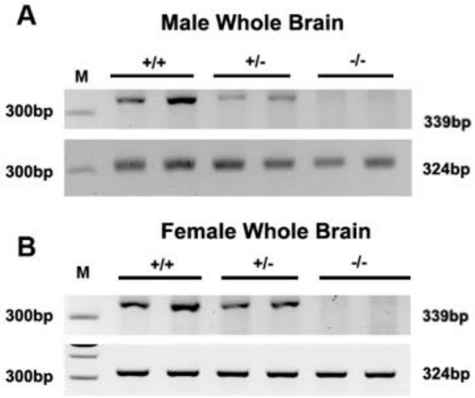


**Supple Fig. 2 RT-PCR analysis of Sam3 mRNA expression from both male (A) and female (B) whole brains of Sam3$^{+/+}$, Sam3$^{+/-}$ and Sam3$^{-/-}$. Male and female SAM3 mRNA expression from the brains of wild-type mice, and heterozygous and homozygous mutant mice.**

## qRT-PCR

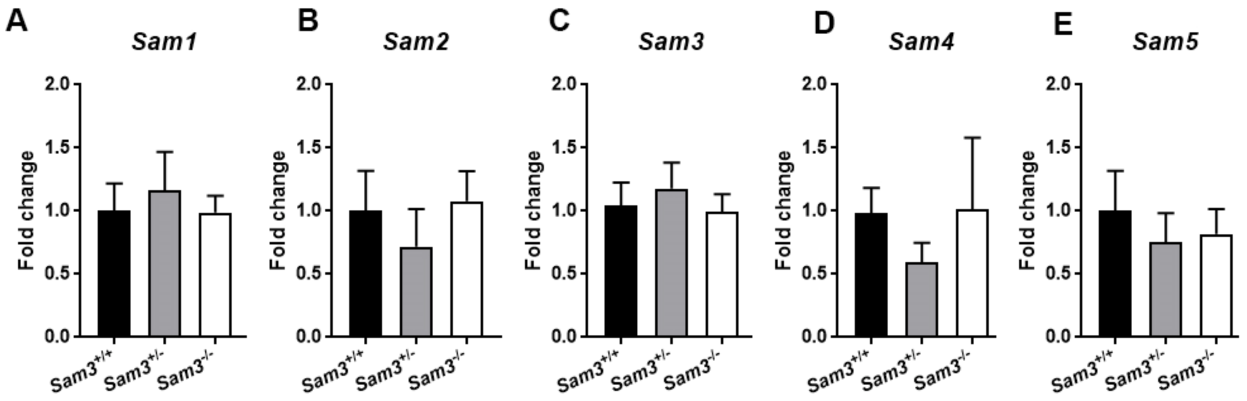


**Supple Fig. 3 qRT-PCR analysis of five Sam family genes in *Sam3*$^{-/-}$ mouse brain.**

(A) *Sam1*. One-way ANOVA with Bonferroni's post hoc test, $F(2,12)=0.1699$, $p=0.8457$

(B) *Sam2*. One-way ANOVA with Bonferroni's post hoc test, $F(2,6)=0.3786$, $p=0.7001$

(C) *Sam3*. One-way ANOVA with Bonferroni's post hoc test, $F(2,12)=0.2847$, $p=0.7571$

(D) *Sam4*. One-way ANOVA with Bonferroni's post hoc test, $F(2,12)=0.4171$, $p=0.6681$

(E) *Sam5*. One-way ANOVA with Bonferroni's post hoc test, $F(2,12)=0.1111$, $p=0.3610$
